# Supplementary material for: Genetic, individual, and familial risk correlates of brain network controllability in major depressive disorder
Source: Mol Psychiatry. 2023 Jan 13;28(3):1057–63. doi: 10.1038/s41380-022-01936-6 (PMC10005934; doi:10.1038/s41380-022-01936-6)

Supplementary Material for

Genetic, Personal, and Familial Risk Correlates of Brain Network Controllability in Major Depressive Disorder

**Key words:** Network Control Theory; diffusion magnetic resonance imaging, structural connectivity; Major Depressive Disorder

**Supplementary Methods**

# Supplementary M1. Genotyping, quality control, and imputation.

Genotyping was conducted using the Infinium PsychArray BeadChip, as described previously.[1] The quality control (QC) of genetic data was conducted in PLINK v1.90b6.10[2] and R v3.5.2, as described previously.[3] Pre-imputation QC of genotype data consisted of the following steps:

1. Removal of SNPs with call rates <98% or a minor allele frequency (MAF) <1%
2. Removal of individuals with genotyping rates <98%
3. Removal of sex mismatches
4. Removal of genetic duplicates
5. Removal of cryptic relatives with pi-hat≥12.5
6. Removal of genetic outliers with a distance from the mean of >4 SD in the first eight multidimensional scaling (MDS) ancestry components
7. Removal of individuals with a deviation of the autosomal or X-chromosomal heterozygosity from the mean >4 SD
8. Removal of non-autosomal variants
9. Removal of SNPs with call rates <98% or a MAF <1% or Hardy-Weinberg Equilibrium (HWE) test *p*-values <1×10^-6^
10. Removal of A/T and G/C SNPs
11. Update of variant IDs and positions to the IDs and positions in the 1000 Genomes Phase 3 reference panel
12. Alignment of alleles to the reference panel
13. Removal of duplicated variants and variants not present in the reference panel

For the calculation of ancestry components (used to determine genetic outliers and as covariates in the analyses), pre-imputation genotype data were used. Additional variant filtering steps were: removal of variants with a MAF <0.05 or HWE *p*‑value <10^‑3^; removal of variants mapping to the extended MHC region (chromosome 6, 25-35 Mbp) or to a typical inversion site on chromosome 8 (7‑13 Mbp); linkage disequilibrium (LD) pruning (command --indep-pairwise 200 100 0.2). Next, the pairwise identity-by-state (IBS) matrix of all individuals was calculated using the command ‑‑genome on the filtered genotype data. Multidimensional scaling (MDS) analysis was performed on the IBS matrix using the eigendecomposition-based algorithm in PLINK v1.90b6.10.

After imputation, variants with a MAF <1%, an HWE test p<1×10-6, and an INFO metric <0.8 were removed. Imputation was conducted using SHAPEIT v2 (r837)[4], IMPUTE2 v2.3.2[5, 6], and the 1000 Genomes Phase 3 reference panel.

In total, imputed genetic data were available for 2,248 individuals.

Variants before QC: 596,861; variants after QC: 284,691; variants after imputation: 8,565,143.

# Supplementary M2. Calculation of polygenic scores.

Two PGSs were calculated using training summary statistics from published genome-wide association studies (GWASs): psychiatric cross-disorder (CD)[7] with 162,151 cases and 276,846 controls and MDD[8] (without 23andMe) with 59,851 cases and 113,154 controls.

PGSs were calculated using the PRS-CS[9] method that employs Bayesian regression to infer PGS weights while modeling local linkage disequilibrium (LD) patterns using the 1000 Genomes EUR reference panel. All training GWAS variants with an INFO metric <0.6, a MAF <1%, or which were not present in the FOR2107 imputation were removed before estimating PRS-CS weights. The global shrinkage parameter was determined using the automatic method. PGSs were calculated in *R* using the PRS-CS weights and imputed dosage data, as described previously[10].

# Supplementary M3. Calculation of polygenic scores.

To estimate medication load, we calculated an established Medication Load Index [11–13], which reflects dose and number of prescriptions irrespective of active components. Each psychotropic medication was coded as absent=0, low=1 (equal or lower average dose), or high=2 (greater than average dose), relative to the midpoint of the daily dose range recommended by Physician’s-Desk-Reference. We calculated a composite measure of total medication load for each individual, reflecting dose and variety of different medications taken, by summing all individual medication.

References

1. Meller T, Schmitt S, Stein F, Brosch K, Mosebach J, Yüksel D, et al. Associations of schizophrenia risk genes ZNF804A and CACNA1C with schizotypy and modulation of attention in healthy subjects. Schizophrenia Research. 2019;208:67–75.

2. Chang CC, Chow CC, Tellier LCAM, Vattikuti S, Purcell SM, Lee JJ. Second-generation PLINK: rising to the challenge of larger and richer datasets. GigaScience. 2015;4.

3. Andlauer TFM, Buck D, Antony G, Bayas A, Bechmann L, Berthele A, et al. Novel multiple sclerosis susceptibility loci implicated in epigenetic regulation. Science Advances. 2016;2:e1501678–e1501678.

4. Delaneau O, Zagury JF, Marchini J. Improved whole-chromosome phasing for disease and population genetic studies. Nature Methods. 2013;10:5–6.

5. Howie B, Fuchsberger C, Stephens M, Marchini J, Abecasis GR. Fast and accurate genotype imputation in genome-wide association studies through pre-phasing. Nature Genetics. 2012;44:955–959.

6. Howie BN, Donnelly P, Marchini J. A Flexible and Accurate Genotype Imputation Method for the Next Generation of Genome-Wide Association Studies. PLoS Genetics. 2009;5:e1000529.

7. Lee PH, Anttila V, Won H, Feng YCA, Rosenthal J, Zhu Z, et al. Genomic Relationships, Novel Loci, and Pleiotropic Mechanisms across Eight Psychiatric Disorders. Cell. 2019;179:1469-1482.e11.

8. Wray NR, Ripke S, Mattheisen M, Trzaskowski M, Byrne EM, Abdellaoui A, et al. Genome-wide association analyses identify 44 risk variants and refine the genetic architecture of major depression. Nature Genetics. 2018;50:668–681.

9. Ge T, Chen C-Y, Ni Y, Feng Y-CA, Smoller JW. Polygenic prediction via Bayesian regression and continuous shrinkage priors. Nature Communications. 2019;10:1776.

10. Andlauer TFM, Guzman-Parra J, Streit F, Strohmaier J, González MJ, Gil Flores S, et al. Bipolar multiplex families have an increased burden of common risk variants for psychiatric disorders. Molecular Psychiatry. 2019. 2019. https://doi.org/10.1038/s41380-019-0558-2.

11. Opel N, Redlich R, Dohm K, Zaremba D, Goltermann J, Repple J, et al. Mediation of the influence of childhood maltreatment on depression relapse by cortical structure: a 2-year longitudinal observational study. The Lancet Psychiatry. 2019;6:318–326.

12. Redlich R, Almeida JJR, Grotegerd D, Opel N, Kugel H, Heindel W, et al. Brain Morphometric Biomarkers Distinguishing Unipolar and Bipolar Depression. JAMA Psychiatry. 2014;71:1222.

13. Repple J, Meinert S, Grotegerd D, Kugel H, Redlich R, Dohm K, et al. A voxel-based diffusion tensor imaging study in unipolar and bipolar depression. Bipolar Disorders. 2017;19:23–31.

**Supplementary Results**

Supplementary Table S1. Regional average controllability association with chronological age in healthy controls.


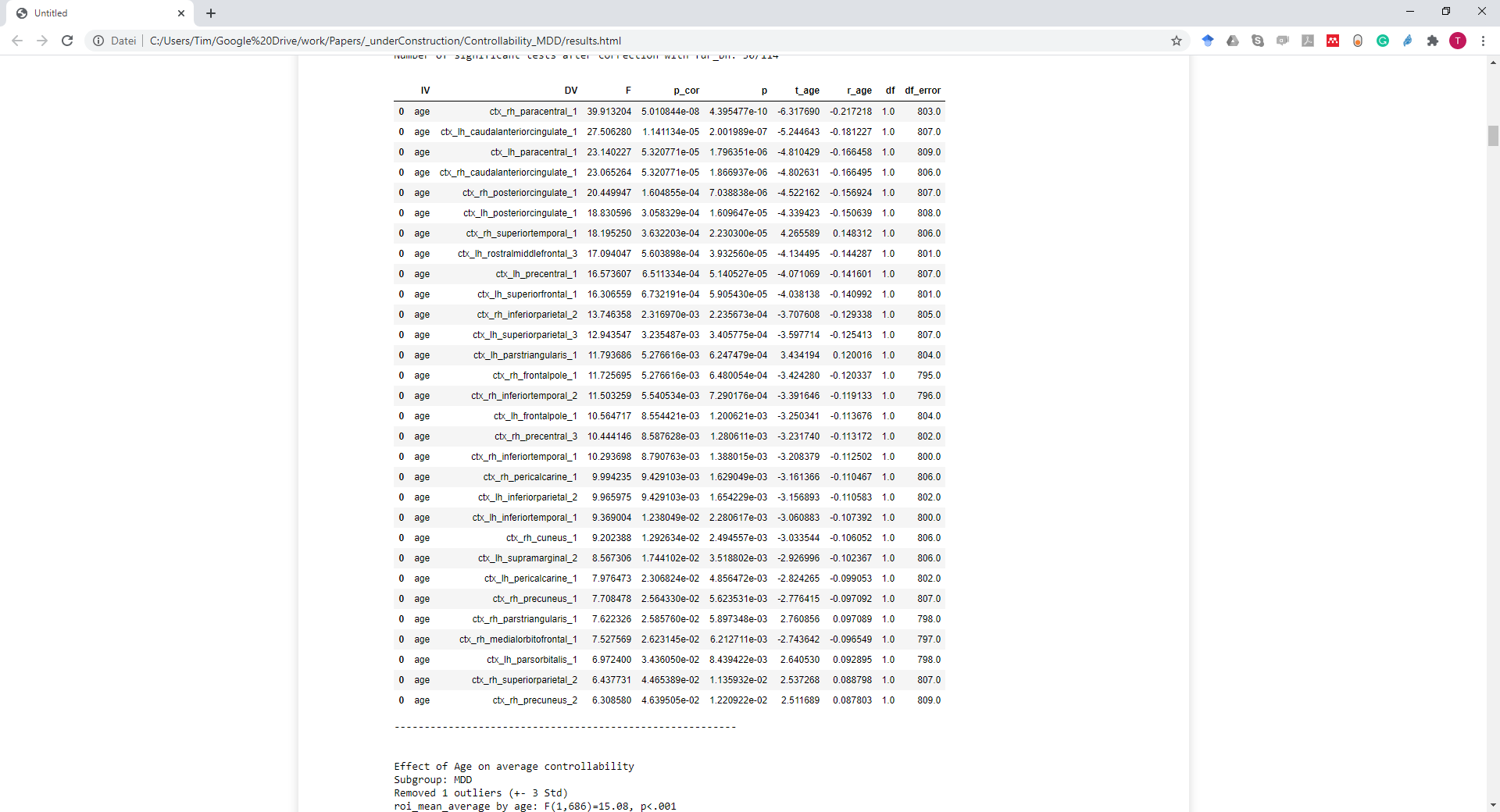


Supplementary Table S2. Regional average controllability association with chronological age in MDD patients.


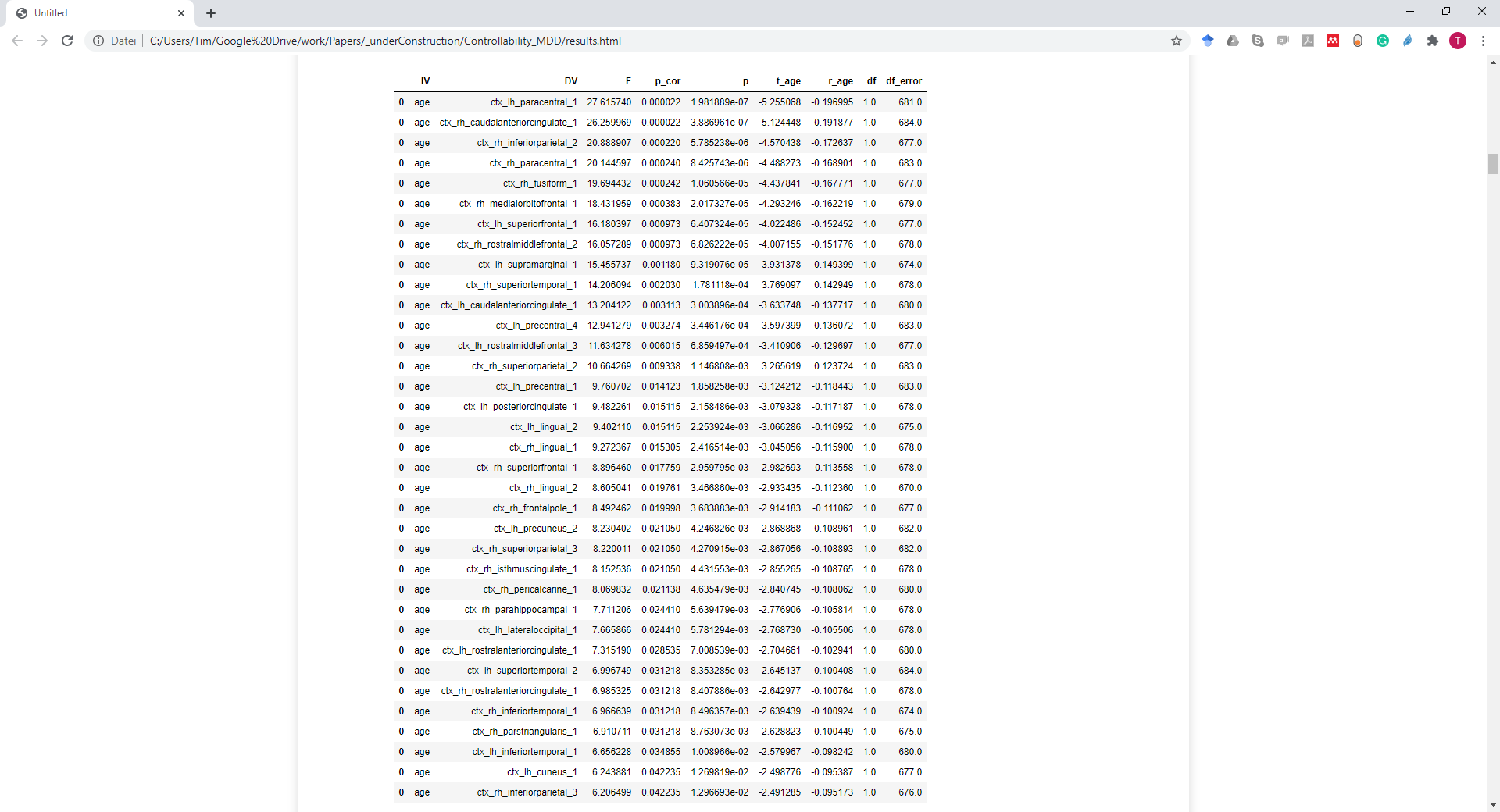


Supplementary Table S3. Regional modal controllability association with chronological age in healthy controls.


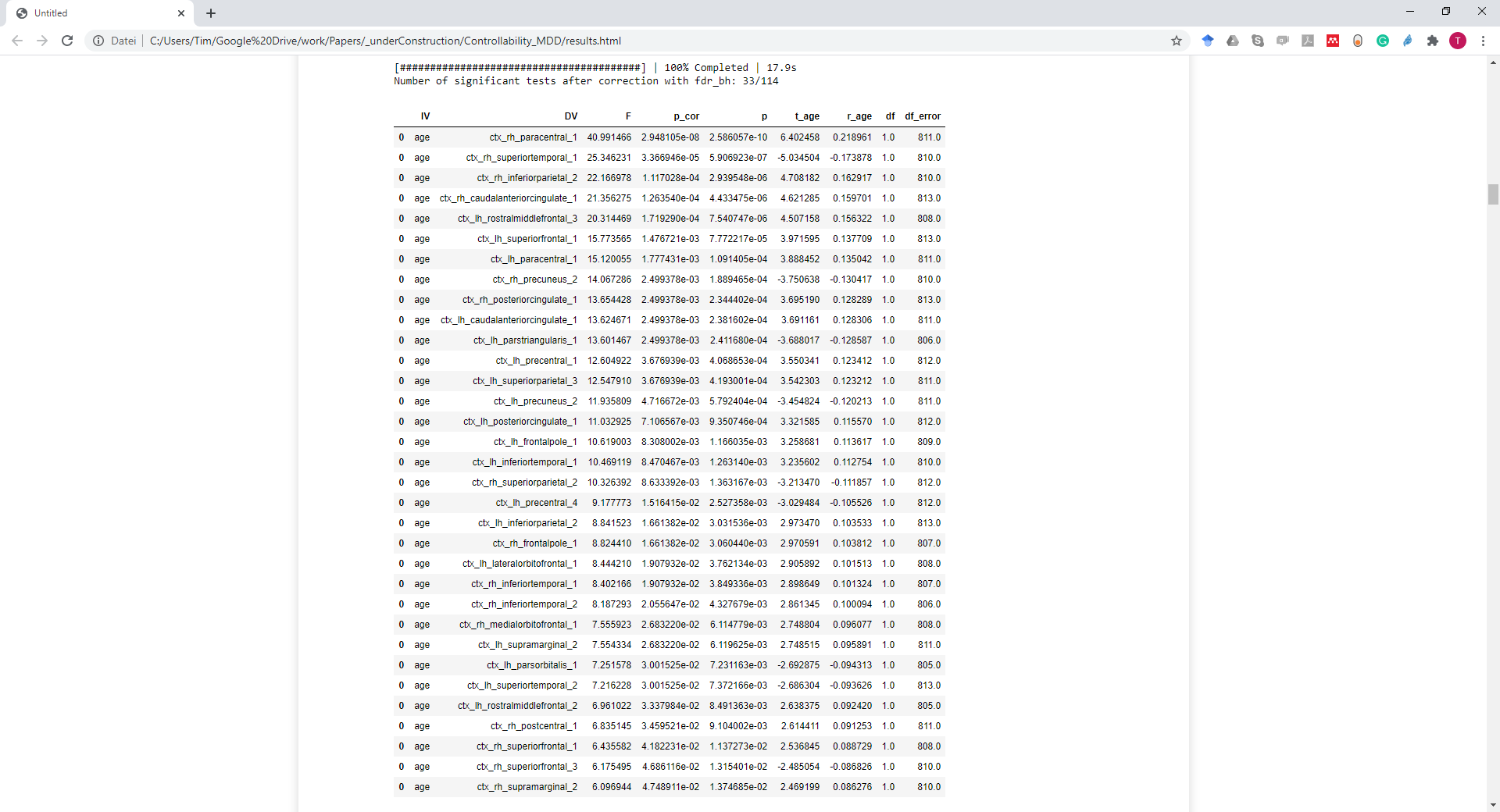


Supplementary Table S4. Regional modal controllability association with chronological age in MDD patients.


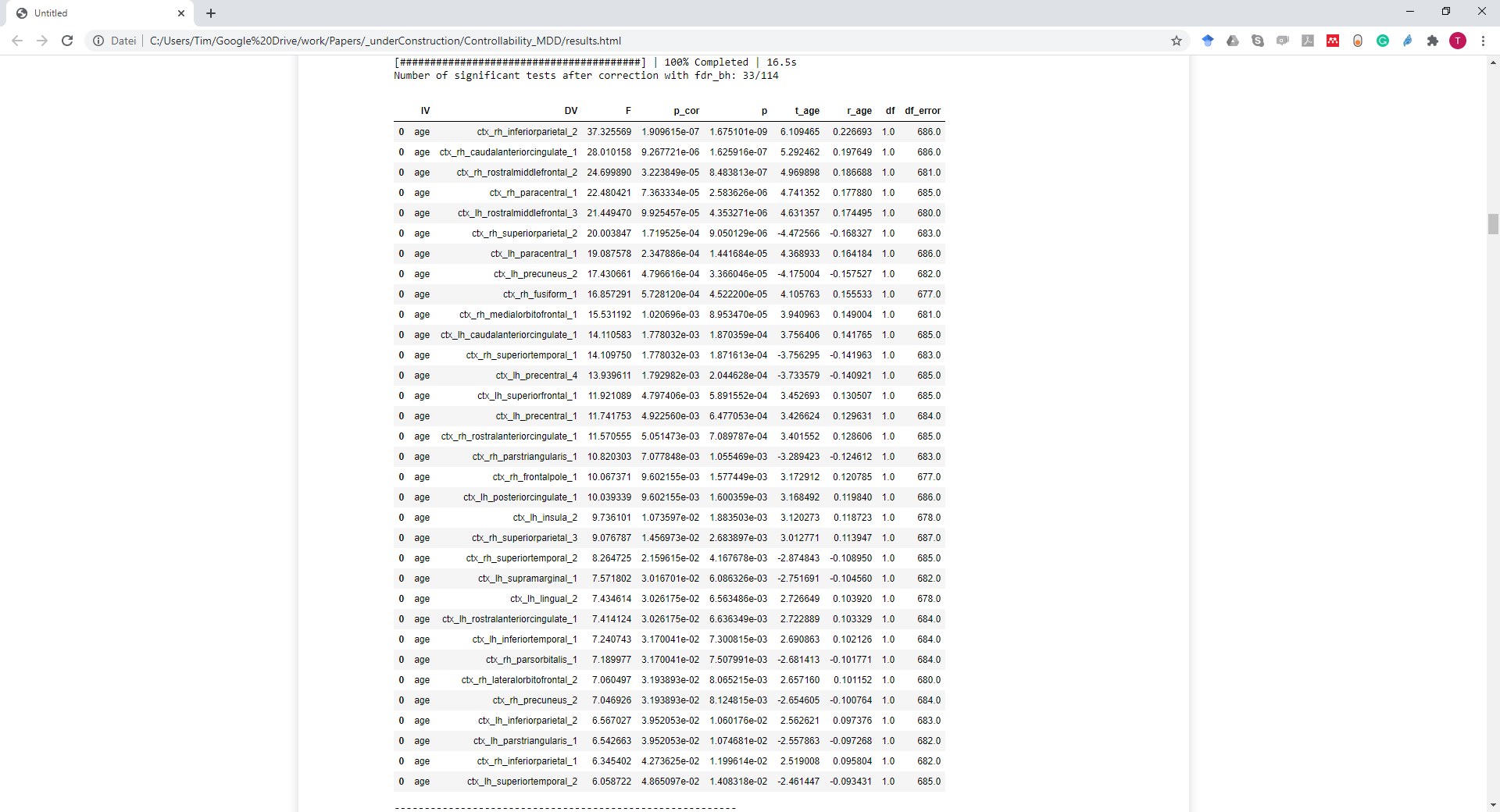


Supplementary Table S5. Regional average controllability association with gender in healthy controls.


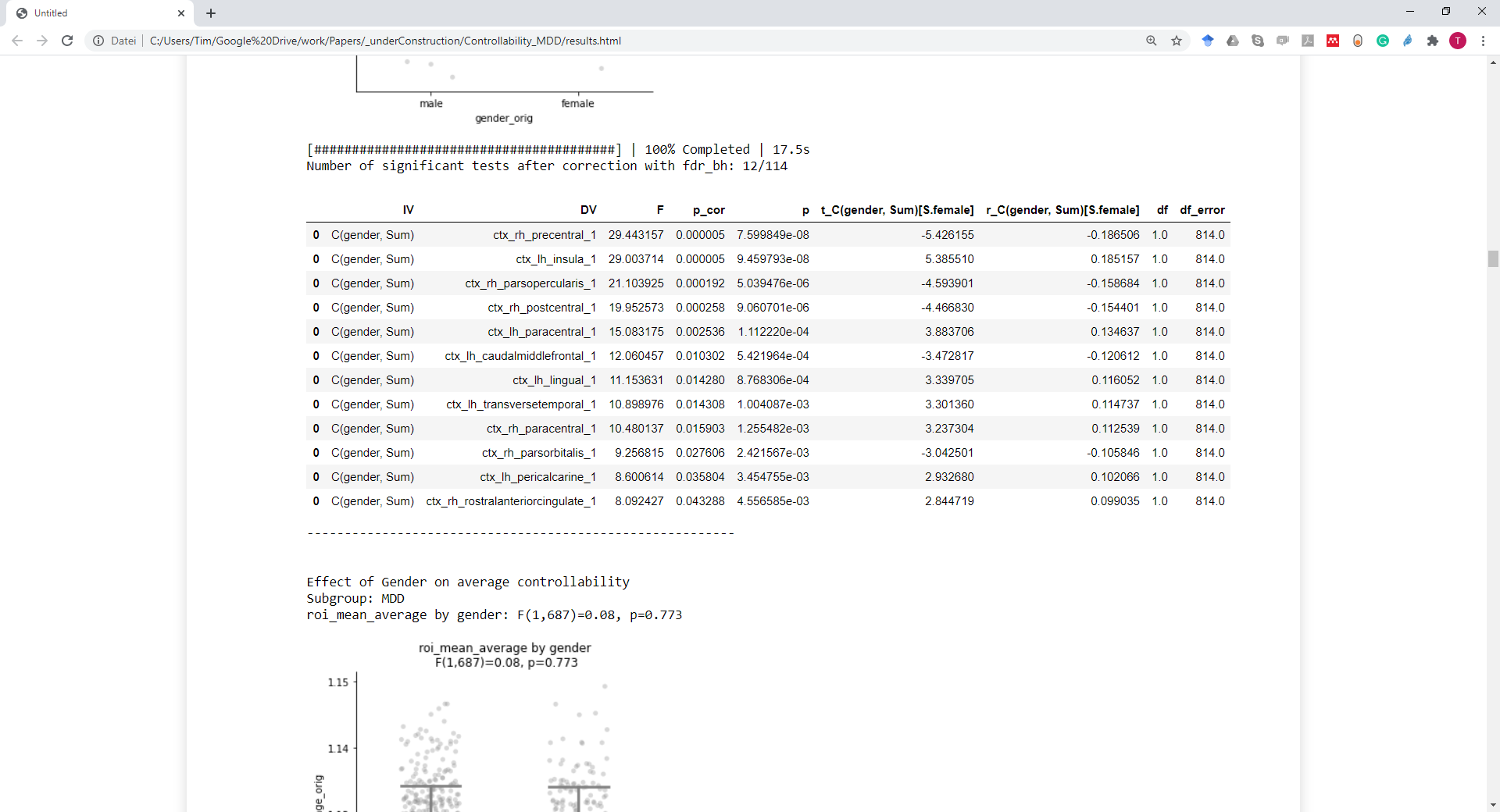


Supplementary Table S6. Regional average controllability association with gender in MDD patients.


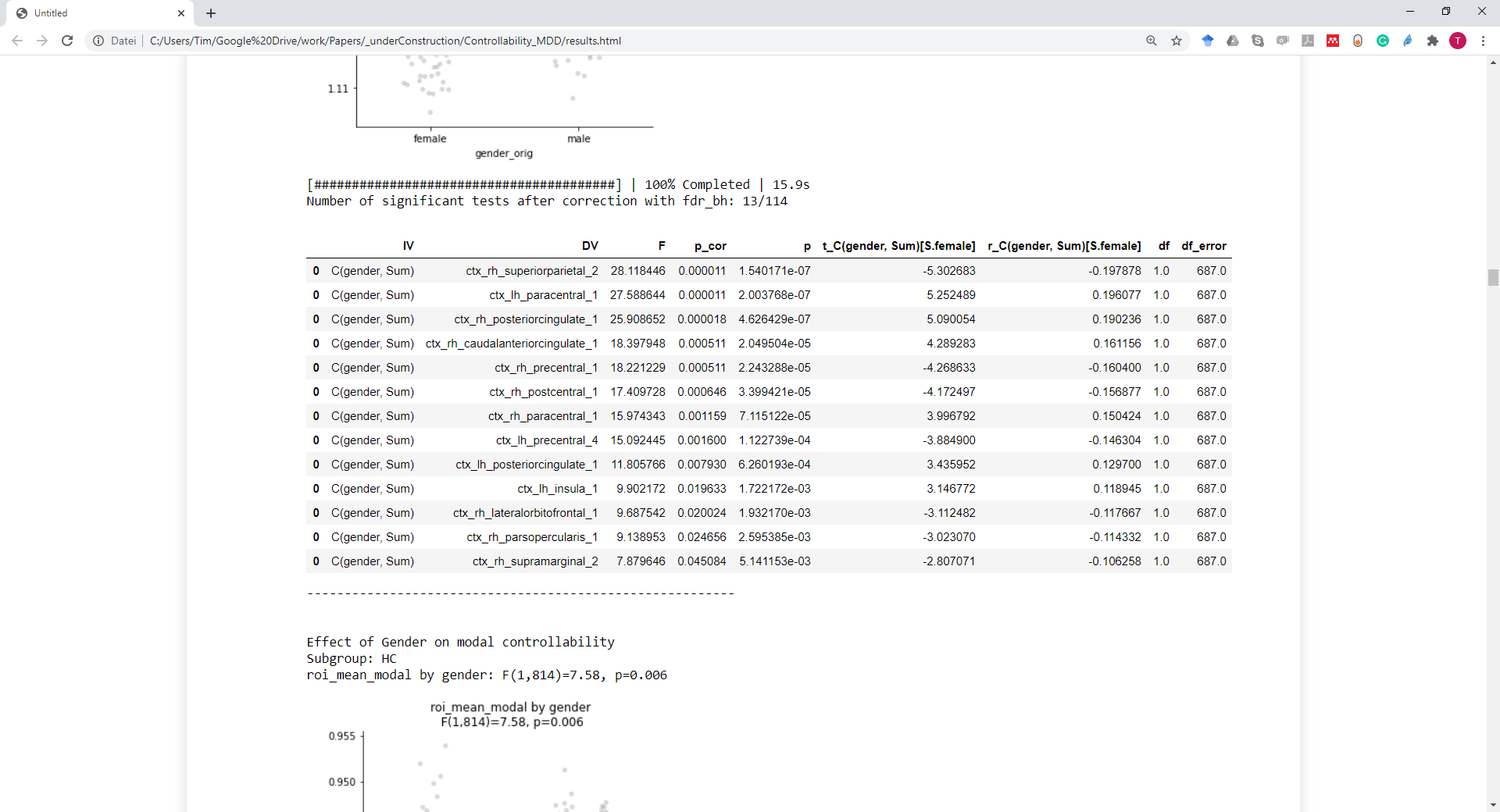


Supplementary Table S7. Regional modal controllability association with gender in healthy controls.


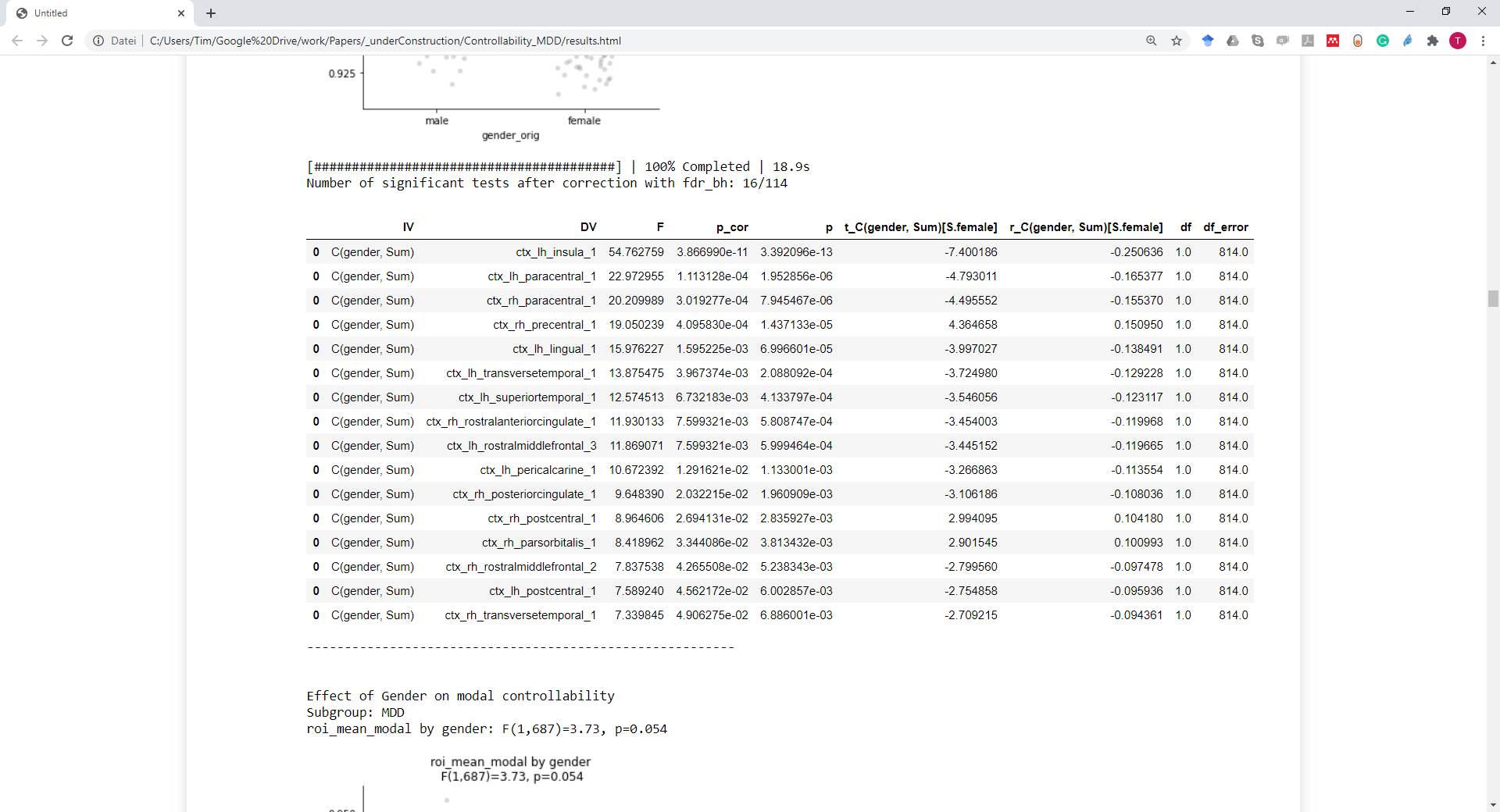


Supplementary Table S8. Regional modal controllability association with gender in MDD patients.


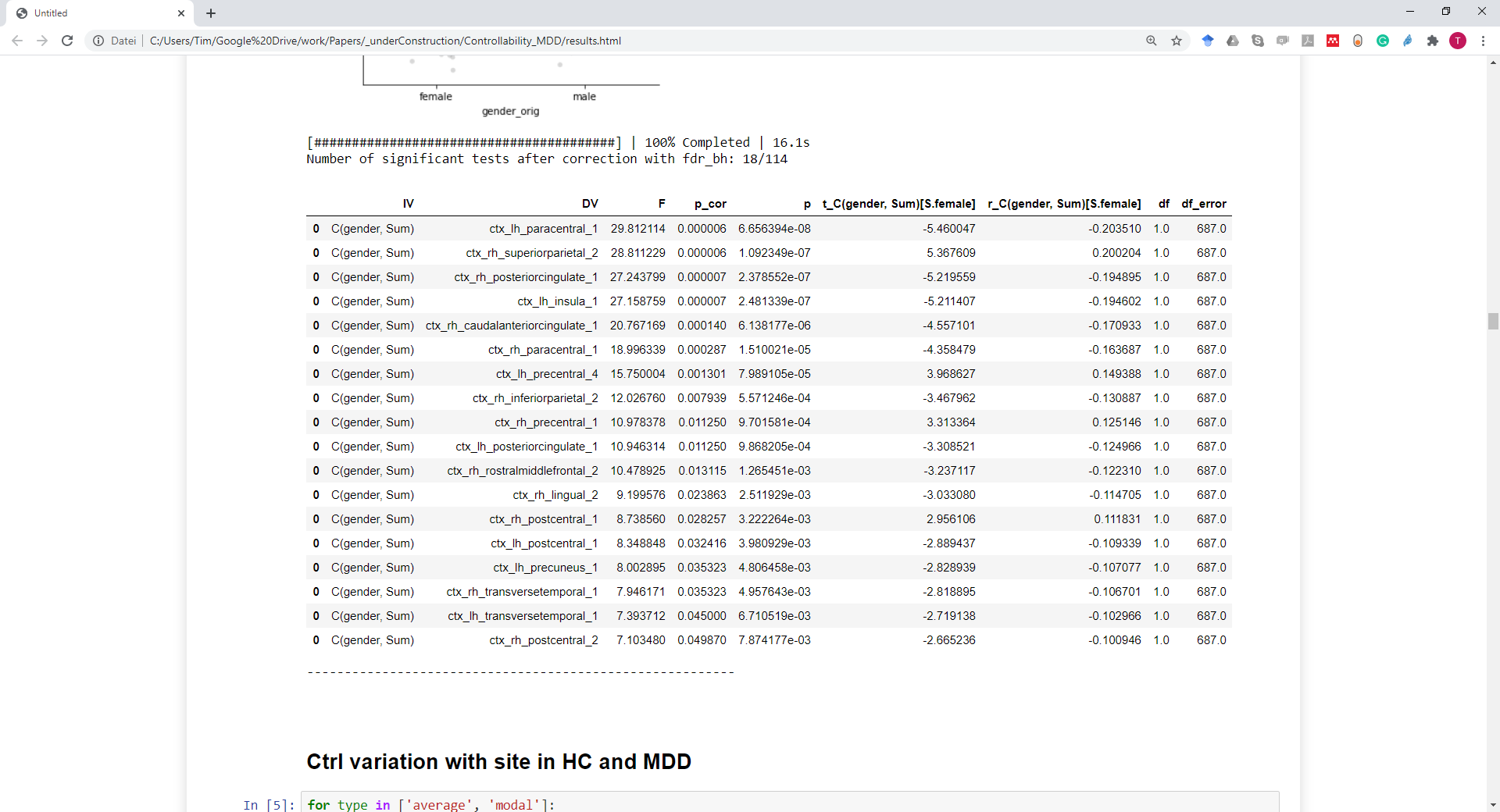


Supplementary Table S9. Regional average controllability association with site in healthy controls.


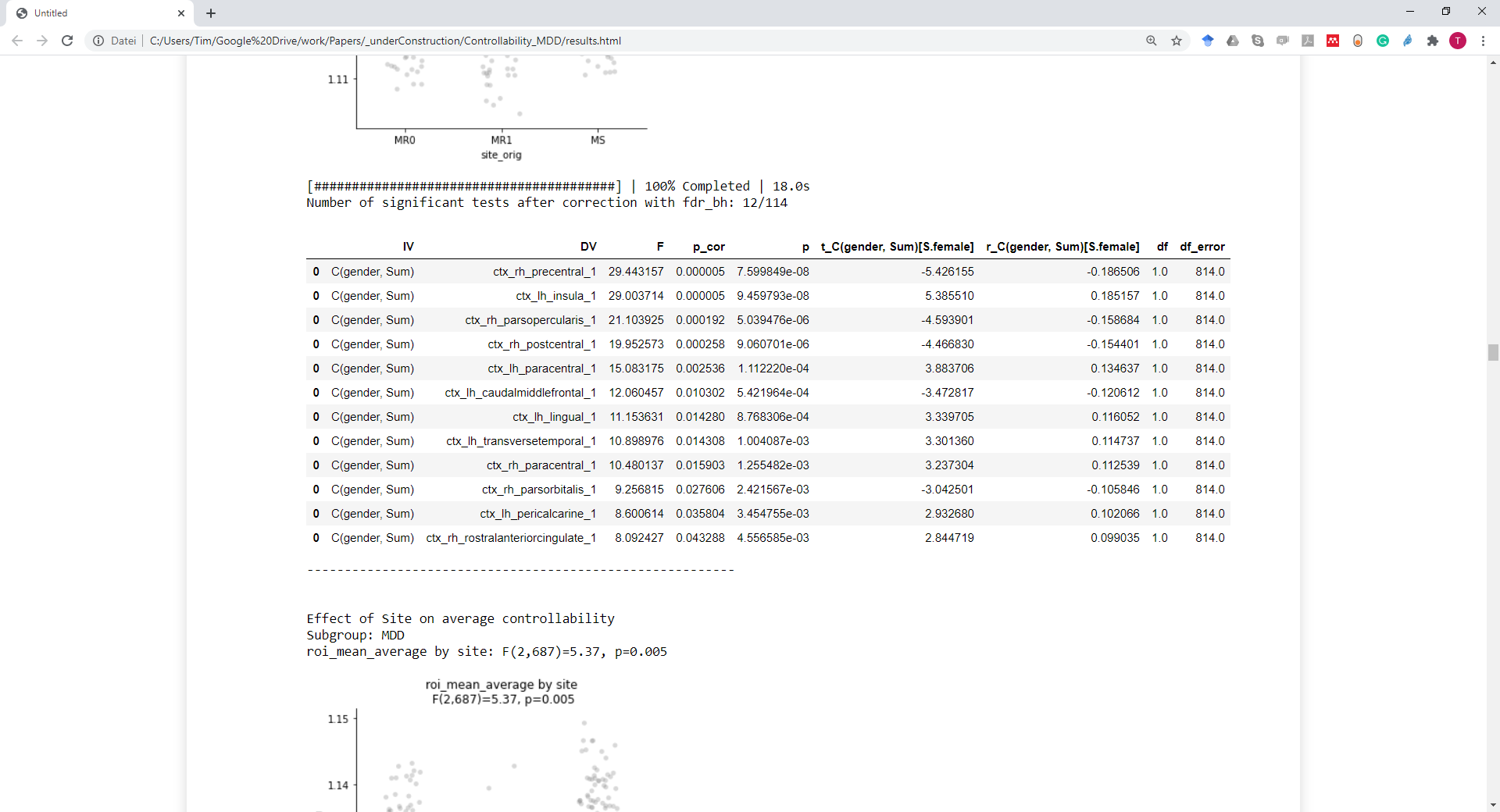


Supplementary Table S10. Regional average controllability association with site in MDD patients.


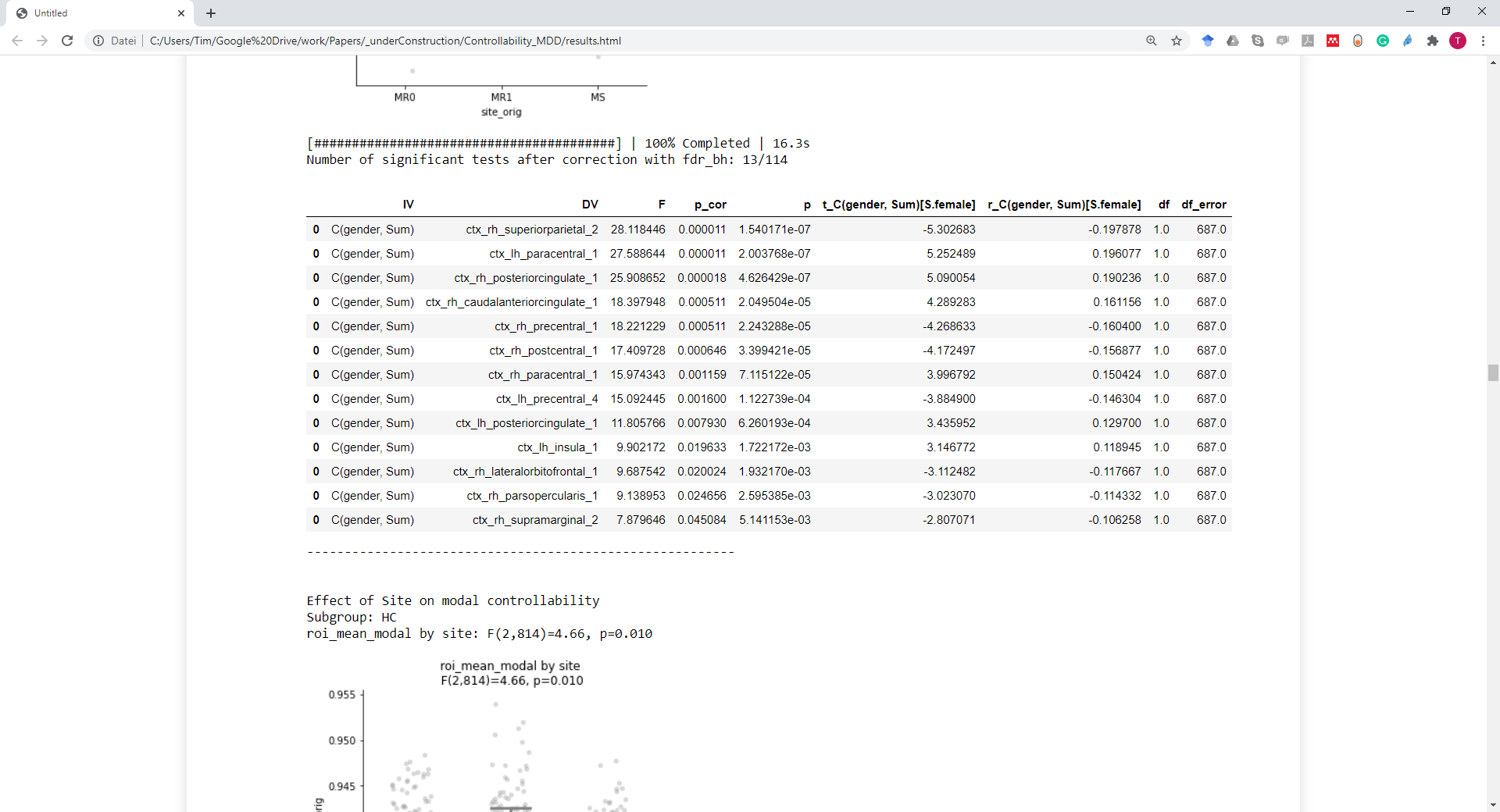


Supplementary Table S11. Regional modal controllability association with site in healthy controls.


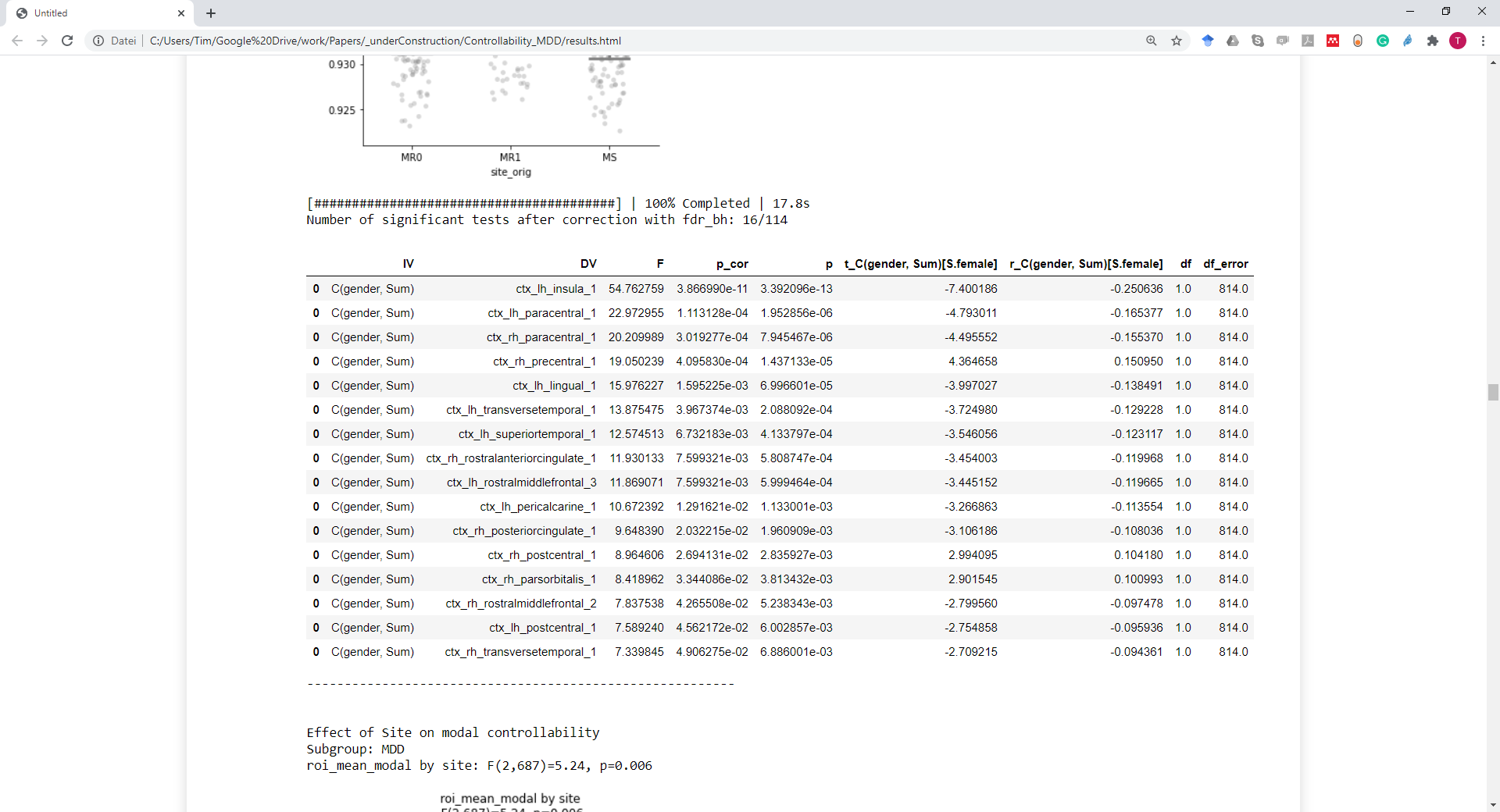


Supplementary Table S12. Regional modal controllability association with site in MDD patients.


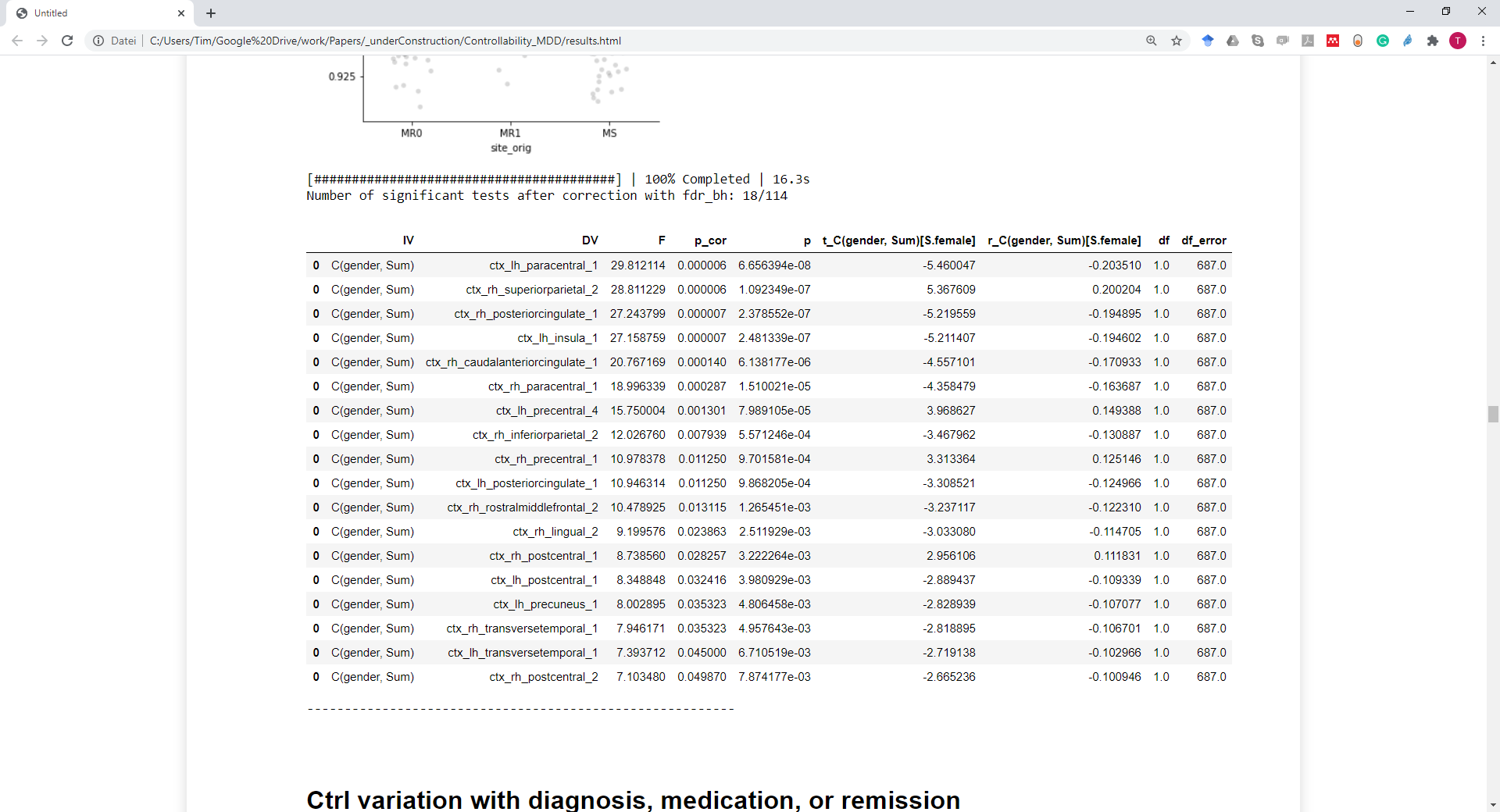


Supplementary Table S13. Regional average controllability association with body mass index in MDD patients.


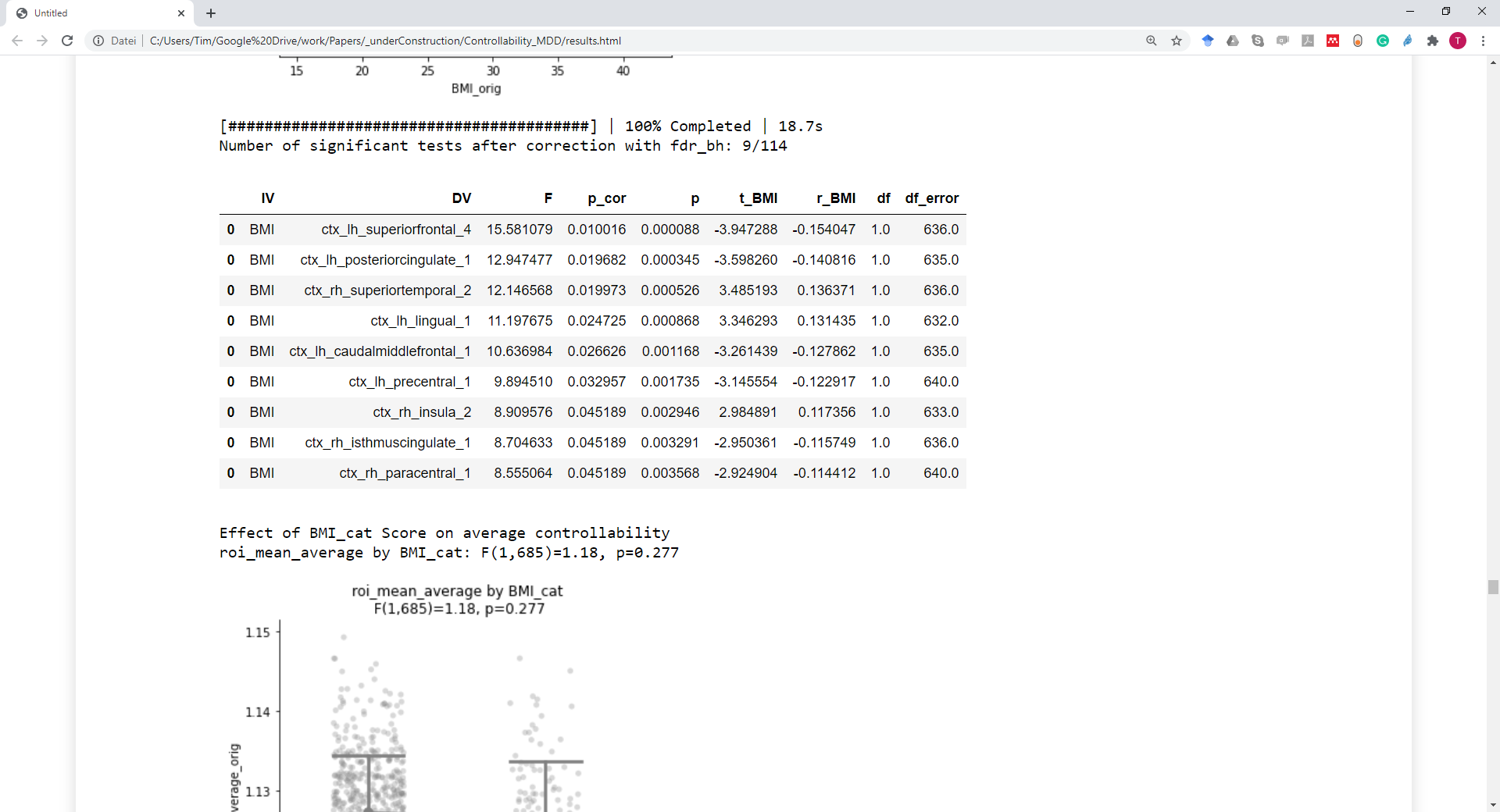


Supplementary Table S14. Regional modal controllability association with body mass index in MDD patients.


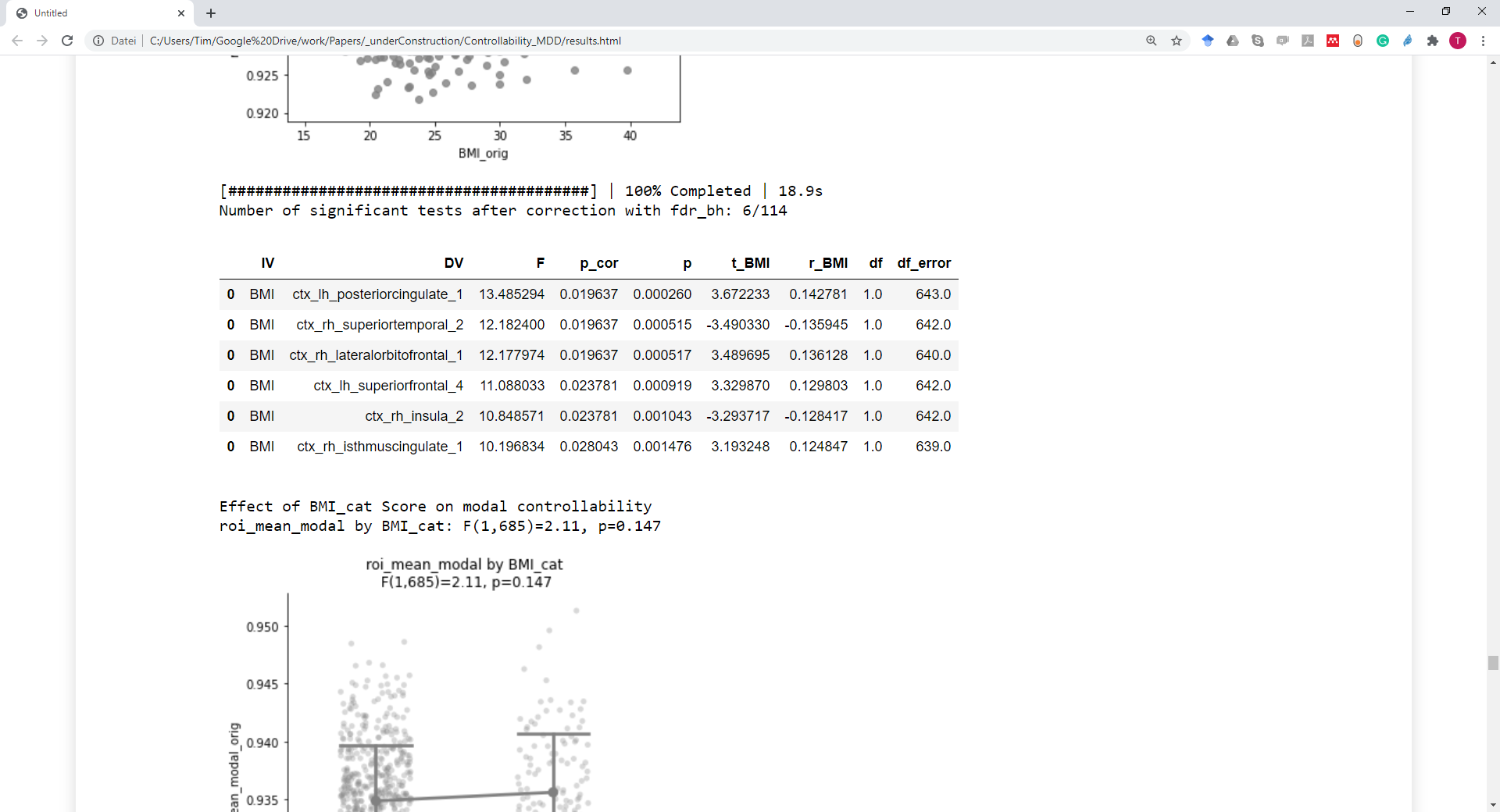


Supplementary Table S15. Regional average controllability association with Familial Risk for Bipolar Disorder in MDD patients.


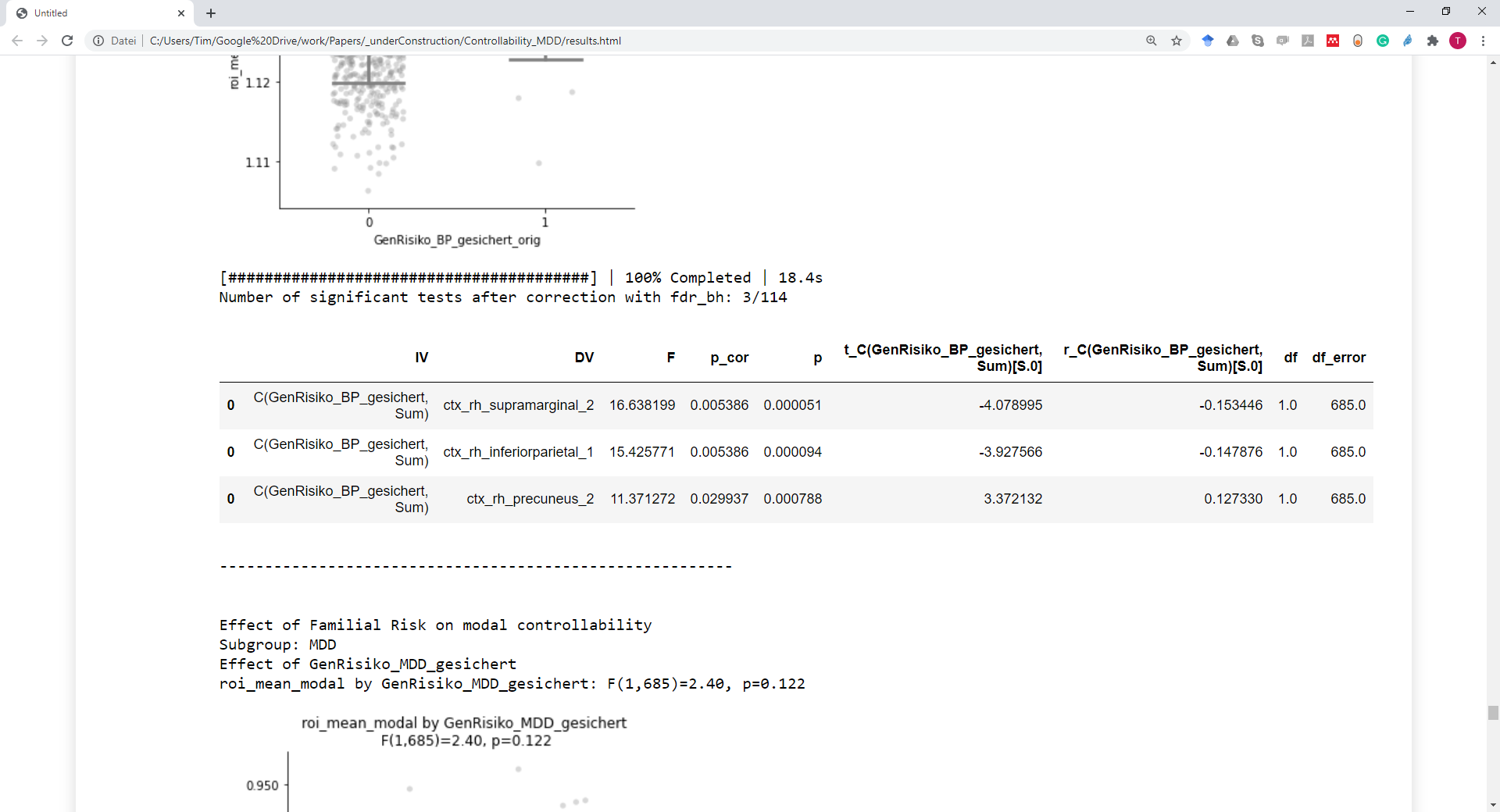


Supplementary Table S16. Regional modal controllability association with Familial Risk for Bipolar Disorder in MDD patients.


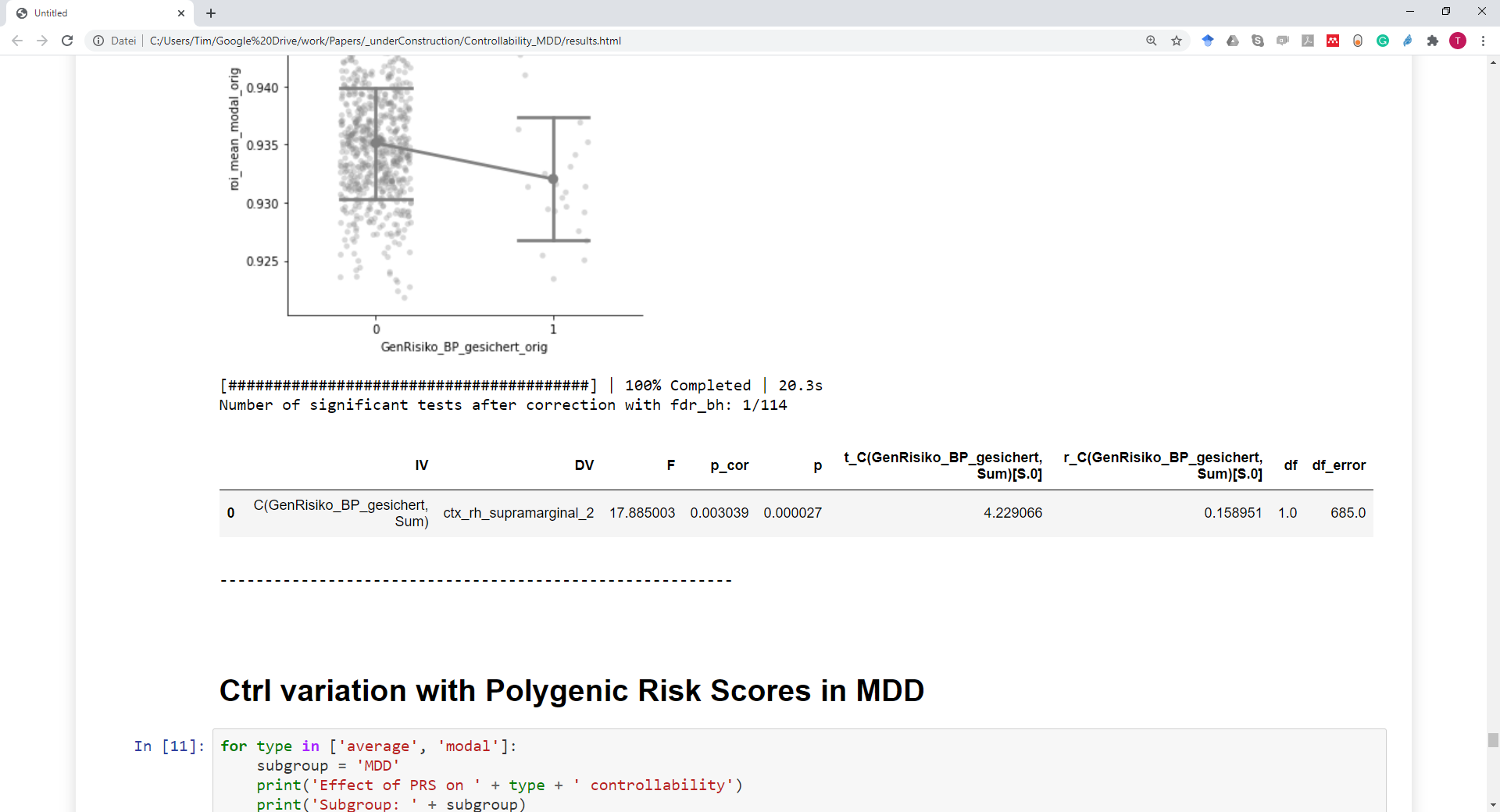

Supplement: Supplementary file 1 — Supplementary Material [file 41380_2022_1936_MOESM1_ESM.docx]
